# Supplementary material for: The influence of investor sentiment on the Chinese stock market amid COVID-19: An event study analysis
Source: PLoS One. 2025 Sep 16;20(9):e0332216. doi: 10.1371/journal.pone.0332216 (PMC12440205; doi:10.1371/journal.pone.0332216)
Supplement: Attachment — Submitted filename: Response Correction File.docx [file pone.0332216.s002.docx]

Author’s Response File

The Influence of Investor Sentiment on the Chinese Stock Market amid COVID-19: An Event Study Analysis

Review Comments to the Author

Please use the space provided to explain your answers to the questions above. You may also include additional comments for the author, including concerns about dual publication, research ethics, or publication ethics. (Please upload your review as an attachment if it exceeds 20,000 characters).

**Author Reply:** Thank you for your valuable time and kind suggestion. Please have a kind review.

Reviewer #1: Comment 1: The introduction covers various aspects of financial crises and the impact of COVID-19, it does not clearly articulate the specific research gap that this paper aims to address. What new insights or contributions will this study provide that have not already been covered by prior work? A stronger statement of the research objective and the gap in existing literature is necessary.

**Author Reply:** Corrections made and introduction highlighted. Thank you for your valuable time and kind suggestion. Please have a kind review.

Comment 2: Terms like “tail-risk,” “Value at Risk (VaR),” and “Conditional Value at Risk (CoVaR)” are introduced without enough context for a non-specialist reader. While the terms may be relevant, they should be explained more clearly to ensure the audience fully understands their importance and how they will be used in the study. A more gradual introduction to complex concepts might be useful, especially for readers unfamiliar with advanced financial models.

**Author Reply:** Corrections made and highlighted. Thank you for your valuable time and kind suggestion. Please have a kind review.

Comment 3: The criteria for sample selection (excluding ST or PT labeled samples, financial sector firms, and those with negative net assets) are clear and aimed at ensuring high data quality. However, the reasons for excluding the financial sector are not discussed. Given the importance of the financial sector in the market, this exclusion should be justified more thoroughly.

**Author Reply:** Corrections made and sample criteria is highlighted in section 3.2 Thank you for your valuable time and kind suggestion. Please have a kind review.

Comment 4: The GubaSenti tool and its origin are well-described. However, a more detailed explanation of how GubaSenti operates and its specific methodologies for sentiment analysis would be beneficial. Additionally, while it is noted that the tool’s reliability and validity were established in previous studies, a brief summary of these studies or their key findings could enhance the credibility of the tool’s application in this research.

**Author Reply:** Corrections made and highlighted in methodology and results. Thank you for your valuable time and kind suggestion. Please have a kind review.

Comment 5: The conclusion asserts that the research contributes to understanding the impact of COVID-19 on the Chinese stock market by highlighting the role of mood in market instability. While this is a valuable insight, the statement could benefit from more explicit connections to existing literature. How does this finding specifically add to or challenge previous research on market reactions to pandemics?

**Author Reply:** Corrections made and introduction section is highlighted in last para. Green highlighted. Thank you for your valuable time and kind suggestion. Please have a kind review.

Comment 6: The recommendation for investors to focus on pharmaceutical stocks during the early stages of the pandemic and to adjust their holdings based on government priorities is practical. However, the advice should be supported by detailed analysis. How were these recommendations derived from the research findings? Including specific data or examples would strengthen the credibility of these investment strategies.

**Author Reply:** Corrections made. Please refer to other tables as well. Thank you for your valuable time and kind suggestion. Please have a kind review.

Reviewer #2: Comments
1.The background of this study is weak, and the introduction section has not been motivated. The authors should highlight the innovation of this study.

**Author Reply:** Corrections made. Please review the introduction section. Thank you for your valuable time and kind suggestion. Please have a kind review.

2.While the contents in the opening remark focus on tail risks and spillover effects, the whole results and discussion seem to deviate from these arguments. The consistency, across the introductory part, literature review, results and discussion, and concluding remarks, could be improved.

**Author Reply:** Corrections made. Please review the literature review section and other content. Highlighted for review. Thank you for your valuable time and kind suggestion. Please have a kind review.

3.On a related note, some irrelevant and duplicated arguments and paragraphs are noticeable.

**Author Reply:** Corrections made. Thank you for your valuable time and kind suggestion. Please have a kind review.

4.The adversarial effects of COVID-19 on stock markets have been intensively investigated in previous empirical studies. What are the contributions and innovations of this study? The authors should further accentuate and clarify the novelty of approaches, contents, and datasets used in this study.

**Author Reply:** Corrections made and introduction section is highlighted in last para. Green highlighted. Thank you for your valuable time and kind suggestion. Please have a kind review.

5.The data section should incorporate more information, including the rationale for selecting the Chinese stock market and data period, data frequency, and criteria for dividing firms into sectors.

**Author Reply:** Corrections made please see the relevant section in the methodology for review. Thank you for your valuable time and kind suggestion. Please have a kind review.

6.The sentiment analysis needs to clarify which datasets are used in identifying investors’ sentiments. Also, what are the results derived from GubaSenti tool? A clear specification of the approach, GubaSenti tool, could improve potential readers’ understandability.

**Author Reply:** Corrections made and irrelevant points removed from that. Thank you for your valuable time and kind suggestion. Please have a kind review. Highlighted for review.

7.What is the “event” introduced in this study? As a key experimental setting, this should be further delineated. All the variables and model specifications need to be further clarified.

**Author Reply:** Event is COVID-19 Outbreak. Corrections made for review. Green highlighted. Thank you for your valuable time and kind suggestion. Please have a kind review.

8.The discussion section narratively lists the numerical results and statistics with few interpretations, falling short of delivering informative insights. The interpretation of findings could be improved by comparing them to existing empirical evidence.

**Author Reply:** See section 5 correction made. Green highlighted. Thank you for your valuable time and kind suggestion. Please have a kind review.

9.The authors could focus on the results of the “Event window” to accentuate the adversarial effects of COVID-19 on stock markets.

**Author Reply:** Corrections made and irrelevant points removed from that. Thank you for your valuable time and kind suggestion. Please have a kind review. Highlighted for review.

10.The authors should have endeavored to remedy the endogenity issues; however, only a limited elaboration is provided.

**Author Reply:** Corrections made Table 5 is added as endogenity table. Thank you for your valuable time and kind suggestion. Please have a kind review. Highlighted for review.

11.Provide the limitations and implications which can be derived from the unique findings of this study.

**Author Reply:** Corrections made. Please review said section. Green marked for review. Thank you for your valuable time and kind suggestion. Please have a kind review.
