# Supplementary material for: The influence of investor sentiment on the Chinese stock market amid COVID-19: An event study analysis
Source: PLoS One. 2025 Sep 16;20(9):e0332216. doi: 10.1371/journal.pone.0332216 (PMC12440205; doi:10.1371/journal.pone.0332216)
Supplement: Attachment — Submitted filename: Response to Reviewers.docx [file pone.0332216.s003.docx]

Dear Reviewers,

We sincerely thank you and the reviewer for taking the time to review our manuscript and for providing constructive comments. We appreciate your positive evaluation of the scientific quality of our work. We have carefully revised the manuscript according to your suggestions, and we believe these revisions have improved its clarity and readability. Below, we provide a detailed response to each comment.

Academic Editor’s Comments

Comment:

Thank you for your revision; a minor revision is requested to improve the overall English quality, particularly focusing on fluency, clarity, grammar, and consistent, precise word choice.

Response:

We have carefully proofread the entire manuscript for grammar, clarity, and consistent word choice. All linguistic inconsistencies and awkward phrasings have been corrected to improve the fluency and readability of the text. A professional language editing service was also used to ensure the manuscript meets publication standards.

Reviewer #1 Comments

Comment 1:

The authors have adequately addressed the previous comments and made substantial improvements to the theoretical framework, hypotheses development, and conceptual clarity. The revised manuscript reflects a clearer distinction between constructs and presents stronger theoretical justifications for the proposed relationships.

However, before the manuscript can be considered for publication, I recommend that the authors conduct a thorough language and grammatical review. Several sections still contain minor linguistic inconsistencies and awkward phrasings that may affect the overall readability and professionalism of the paper.

Response:

We sincerely thank the reviewer for the positive evaluation of our revisions. In accordance with your suggestion, we have conducted a thorough language and grammatical review of the manuscript. All sections have been carefully revised to eliminate inconsistencies and awkward phrasing, ensuring improved readability, clarity, and professionalism.

Comment 2:

Once these minor language issues are addressed, I believe the paper will be suitable for publication.

Response:

We greatly appreciate the reviewer’s acknowledgment. The manuscript has been fully revised for language quality, and we believe it is now ready for publication.

Reference List

If any references were updated, replaced, or corrected, include a brief statement here:

Response:

All references have been reviewed and updated as necessary to ensure accuracy and compliance with PLOS ONE guidelines. No retracted articles are cited; all references are current and relevant.

We thank the editor and reviewer once again for their valuable comments and guidance. We hope that the revisions we have made address all concerns and that the manuscript is now suitable for publication in PLOS ONE.

Sincerely,

Sun
